# Comment on Gaspari et al. Blood Purification in Hepatic Dysfunction after Liver Transplant or Extensive Hepatectomy: Far from the Best-Case Scenarios. *J. Clin. Med.* 2024, *13*, 2853

**DOI:** 10.3390/jcm14030716

**Published:** 2025-01-23

**Authors:** Ivano Riva, Stefano Faenza, Antonio Siniscalchi, Elisabetta Cerutti, Giandomenico Luigi Biancofiore

**Affiliations:** 1 General Intensive Care Unit, Azienda Socio Sanitaria Territoriale Papa Giovanni XXIII, 24127 Bergamo, Italy; 2 Department of Specialist, Diagnostic, and Experimental Medicine, Bologna University, 40138 Bologna, Italy; stefano.faenza@unibo.it; 3 Medical-Surgical Department of Digestive, Hepatic and Endocrine-Metabolic Diseases, Bologna University, 40138 Bologna, Italy; antonio.siniscalchi@aosp.bo.it; 4 Department of Anesthesia, Transplant and Surgical Intensive Care, Azienda Ospedaliero Universitaria Delle Marche, 60126 Ancona, Italy; elisabetta.cerutti@ospedaliriuniti.marche.it; 5 Transplant Anesthesia and Critical Care, University of Pisa Medical School Hospital, Via Paradisa 2, 56124 Pisa, Italy; giandomenico.biancofiore@unipi.it

We read with interest the paper entitled Case Report Blood Purification in Hepatic Dysfunction after Liver Transplant or Extensive Hepatectomy: Far from the Best-Case Scenarios, which was recently published in the Journal of Clinical Medicine [1].

We would like to observe that although achieving its primary endpoint (i.e., a significant reduction in serum total bilirubin at the end of the sorbent treatment), the results for the secondary endpoint deserve some discussion as they contradict our clinical experience and the scientific literature available so far.

First of all, in their secondary endpoint analysis, the authors investigated whether bilirubin levels continued to decrease 72 h after the discontinuation of the sorbent treatment and found that bilirubin serum levels rose again, indicating that the initial reduction in bilirubin achieved with the treatment was not sustained [1]. To better evaluate this result, it would have been necessary to make more data available to readers. In particular, since only two sorbents in five patients, as well as three sorbents in two patients, were used, it would be interesting to know how many hours each sorbent was used for and when it was replaced. Further, why did the authors not perform pre-/post-sorbent measurements of bilirubin levels to find out when the sorbents became saturated? Moreover, did they decide to stop the sorbent treatment based on the filters’ saturation times or just considering the total bilirubin levels trend over time? Unfortunately, we do not have such information as the authors limited their measurements to the systemic bilirubin levels without performing pre-and post-sorbent blood samplings at different time-points, which would have allowed for the calculation of the mass balance (MB), which is the only parameter capable of returning correct information on the actual effectiveness of the removal, on the possible saturation, and, consequently, on the number of sorbents to be used, as well as on the convenient timing for replacement [2].

In fact, using the pre-/post-sorbent sampling and the MB calculation, the in vitro study conducted by Gemelli et al. [3] has demonstrated that albumin levels remained stable across all experiments; bilirubin adsorption was consistent throughout the entire duration of the study, even at low bilirubin serum concentrations; and no rebound in bilirubin levels was observed. This suggests that bilirubin binds irreversibly to the sorbent, a conclusion that is further supported by the fact that the resin did not release bilirubin when incubated in a fresh albumin solution for 24 h. Although the bilirubin removal in vivo may be affected by biological competition mechanisms, the irreversible binding of bilirubin to the sorbent remains undisputed under any condition.

Therefore, the secondary endpoint results of Gaspari and colleagues’ study appear to be burdened by significant methodological limitations and the observed rise in bilirubin levels 72 h after the sorbent’s discontinuation simply indicate that the adopted therapeutic regimen was insufficient or ineffective in addressing the underlying problem.

Secondly, the authors conclude by suggesting caution in the use of CytoSorb^®^ (Cytosorbents medical Inc., Princeton, NJ, USA) in thrombocytopenic patients with liver failure, due to its possible harmful effects on hemostasis, because of the observed reduction in platelet count and increase in D-dimer (which was attributed to the sorbent’s treatment). However, as the authors themselves state, this observation could be related to several different mechanisms, as follows: (a) the specific characteristics and clinical conditions of the enrolled patients, who may suffer from underlying coagulopathies; (b) variations in anticoagulation protocols used during the sorbent treatment; (c) the continuous renal replacement therapy that can affect coagulation parameters and contribute to the observed D-dimer change. Moreover, the authors’ results contrast with previous studies where an increase was not found but, rather, a decrease [4,5,6,7,8,9,10,11,12,13,14].

Moreover, as for the platelet count reduction, it is well documented that patients with serious illnesses, such as those characterizing the enrolled patients, often have coagulation abnormalities and that any extracorporeal treatment can affect platelet count [15,16]. All of these factors should have been considered in interpreting the study results, which could have been definitively clarified if a control group had been considered. In fact, without a control group where CRRT alone was used, it is very difficult to distinguish the effects of the absorbent treatment from those of CRRT and the underlying disease. Therefore, the authors’ conclusion to exercise caution in using CytoSorb^®^ in thrombocytopenic patients with liver failure because of its possible detrimental effects on hemostasis is hardly understandable. In fact, a decrease in platelet count in the critically ill can be due to several different causes, including the use of heparin for anticoagulation of the extracorporeal circuit or platelets being trapped in extracorporeal circuits and CRRT filters. Finally, we would like to note that several studies have demonstrated non-significant reductions in platelet count without complications due to bleeding from coagulopathy or an increase in transfusion requirements during the sorbent treatment [9,10,11,12,13,14].

In summary, we believe that in addition to the numerous limitations correctly acknowledged by the authors themselves, this paper seems to be affected by several methodological drawbacks and by a partial and limited interpretation of its results.

## References

[1] Gaspari R., Aceto P., Spinazzola G., Piervincenzi E., Chioffi M., Giuliante F., Antonelli M., Avolio A.W. (2024). Blood Purification in Hepatic Dysfunction after Liver Transplant or Extensive Hepatectomy: Far from the Best-Case Scenarios. J. Clin. Med..

[2] Riva I., Marino A., Valetti T.M., Marchesi G., Fabretti F. (2023). Extracorporeal liver support techniques: A comparison. J. Artif. Organs.

[3] Gemelli C., Cuoghi A., Magnani S., Atti M., Ricci D., Siniscalchi A., Mancini E., Faenza S. (2019). Removal of Bilirubin with a New Adsorbent System: In Vitro Kinetics. Blood Purif..

[4] Wagner R., Soucek P., Ondrasek J., Fila P., Sterba J., Spacilova H., Michalcikova A., Freiberger T., Nemec P. (2019). Plasma Levels of Myocardial MicroRNA-133a Increase by Intraoperative Cytokine Hemoadsorption in the Complex Cardiovascular Operation. J. Clin. Med. Res..

[5] Mehta Y., Mehta C., Kumar A., George J.V., Gupta A., Nanda S., Kochhar G., Raizada A. (2020). Experience with hemoadsorption (CytoSorb^®^) in the management of septic shock patients. World J. Crit. Care Med..

[6] Rugg C., Klose R., Hornung R., Innerhofer N., Bachler M., Schmid S., Fries D., Ströhle M. (2020). Hemoadsorption with CytoSorb in Septic Shock Reduces Catecholamine Requirements and In-Hospital Mortality: A Single-Center Retrospective ’Genetic’ Matched Analysis. Biomedicines.

[7] Supady A., Zahn T., Rieder M., Benk C., Lother A., Bode C., Wengenmayer T., Staudacher D., Kellum J.A., Duerschmied D. (2022). Effect of Cytokine Adsorption on Survival and Circulatory Stabilization in Patients Receiving Extracorporeal Cardiopulmonary Resuscitation. ASAIO J..

[8] Mariano F., Greco’ D., Depetris N., Mella A., Sciarrillo A., Stella M., Berardino M., Risso D., Gambino R., Biancone L. (2024). CytoSorb^®^ in burn patients with septic shock and Acute Kidney Injury on Continuous Kidney Replacement Therapy is associated with improved clinical outcome and survival. Burns.

[9] Popescu M., David C., Marcu A., Olita M.R., Mihaila M., Tomescu D. (2023). Artificial Liver Support with CytoSorb and MARS in Liver Failure: A Retrospective Propensity Matched Analysis. J. Clin. Med..

[10] Ocskay K., Tomescu D., Faltlhauser A., Jacob D., Friesecke S., Malbrain M., Kogelmann K., Bogdanski R., Bach F., Fritz H. (2021). Hemoadsorption in ‘Liver Indication’—Analysis of 109 Patients’ Data from the CytoSorb International Registry. J. Clin. Med..

[11] Tomescu D., Popescu M., David C., Sima R., Dima S. (2021). Haemoadsorption by CytoSorb^®^ in patients with acute liver failure: A case series. Int. J. Artif. Organs.

[12] Turan C., Szigetváry C.E., Kói T., Engh M.A., Atakan I., Zubek L., Terebessy T., Hegyi P., Molnár Z. (2023). Hemoad-sorption Therapy for Critically Ill Patients with Acute Liver Dysfunction: A Meta-Analysis and Systematic Review. Biomedicines.

[13] Peri A.M., Magro B., van den Bogaart L., Dalla Pria A., Giuffrida P., Gianatti A., Fabretti F., Barbui A.M., Tebaldi A., Rizzi M. (2021). Successful Treatment of Suspected Donor-derived Human Herpesvirus-8 Infection in a Liver Transplant Patient with Coronavirus Disease-19. Transplantation.

[14] Bruenger F., Kizner L., Al Khali R., Gummert J. (2017). Successful Treatment of Severe Sepsis with Disseminated Intravascular Coagulopathy.

[15] Martucci G., Rossetti M., Li Petri S., Alduino R., Volpes R., Panarello G., Gruttadauria S., Burgio G., Arcadipane A. (2022). Continuous Renal Replacement Therapy after Liver Transplantation: Peri-Operative Associated Factors and Impact on Survival. J. Clin. Med..

[16] Fisher R., Moore G.W., Mitchell M.J., Dai L., Crichton S., Lumlertgul N., Ostermann M. (2022). Effects of regional citrate anticoagulation on thrombin generation, fibrinolysis and platelet function in critically ill patients receiving continuous renal replacement therapy for acute kidney injury: A prospective study. Ann. Intensive Care.

